# Dual-action antimicrobial peptide therapy: disrupting biofilm formation and targeting bacterial DNA for enhanced treatment of *Pseudomonas aeruginosa* endophthalmitis

**DOI:** 10.1128/spectrum.01180-25

**Published:** 2026-05-15

**Authors:** Yi Tang, Yan-Jie Zhou, Zi-Chao Ning, Yue Zhang, Sheng Qu, Nan Li, Zhuo Hao, Zhan-Yuin Ong, Hong Wu

**Affiliations:** 1 Eye Center of Second Hospital, Jilin University 12510, Changchun, China; 2 Changchun Veterinary Research Institute 595703 , Changchun, China; 3 The School of Physics and Astronomy and School of Medicine, University of Leeds 4468, Leeds, United Kingdom; Guizhou Medical University, Guiyang, China

**Keywords:** anti-microbial peptides, endophthalmitis, bacterial biofilm, *Pseudomonas aeruginosa*

## Abstract

Intraocular infections caused by *Pseudomonas aeruginosa* are difficult to treat due to bacterial biofilm-mediated antibiotic resistance. We used cationic polypeptide (XXYY) n to evaluate its effect and mechanism against *Pseudomonas aeruginosa* biofilm and its efficacy against *Pseudomonas aeruginosa* intraocular infection. Microbroth dilution showed that the MIC_90_ of (LLKK)_3_C against *Pseudomonas aeruginosa* was 32 μg/mL. After continuous passage for 10 generations, the stability of antibacterial activity of (LLKK)_3_C against *Pseudomonas aeruginosa* was better than that of amikacin. TEM, SEM and live/dead staining demonstrated that (LLKK)_3_C was able to destroy the integrity of bacterial and biofilm and exert antibacterial activity by interacting with phosphatidyl glycerol on the membrane to destroy the bacterial membrane. Electrophoretic gel and Alphafold3 demonstrated that (LLKK)_3_C binds to bacterial DNA through charge interactions. In the rabbit endophthalmitis model, the intraocular bacterial load decreased after intravitreal injection (LLKK)_3_C treatment (vs untreated, *P* < 0.05). Studies have shown that (LLKK)_3_C has synergistic antibacterial effects through the dual mechanism of targeting membrane destruction and DNA binding, which provides a new treatment strategy for biomembrane-associated intraocular infections.

**IMPORTANCE:**

Ocular infections caused by *Pseudomonas aeruginosa* often lead to severe vision loss due to antibiotic-resistant bacterial biofilms, which shield bacteria from conventional treatments. This study introduces (LLKK)3C, that uniquely attacks biofilms through a dual-action mechanism: physically disrupting bacterial membranes and binding to bacterial DNA. Unlike traditional antibiotics like amikacin, (LLKK)3C maintains robust antibacterial activity even after prolonged exposure, reducing the risk of resistance development. In a rabbit endophthalmitis model, (LLKK)3C significantly reduced intraocular bacterial loads, demonstrating its potential as a targeted therapy for sight-threatening infections. By addressing the dual challenges of biofilm resistance and drug stability, (LLKK)3C offers a promising strategy to improve clinical outcomes for patients with difficult-to-treat eye infections.

## INTRODUCTION

*Pseudomonas aeruginosa* (*P. aeruginosa*) is one of the most prevalent gram-negative (G−) bacteria causing infectious intraocular diseases, which leads to vision loss, even eyeball enucleation (1–3). An outbreak of carbapenem-resistant *P. aeruginosa* strain (VIM-GES-CRPA) in the United States in 2023 contaminated EzriCare artificial tears and caused severe eye infections, affecting at least 81 people and causing 4 deaths (4, 5). A retrospective study of *P. aeruginosa* endophthalmitis from 2002 to 2012 showed that even if all patients received intravitreal vancomycin combined with ceftazidime injection, 67% (*n* = 12) of patients ended up with vision loss and 42% (*n* = 12) enucleation (6). The antimicrobial therapy of *P. aeruginosa* is limited due to its inherent resistance, membrane permeability, efflux pump, biofilm, and lateral gene transfer (7). A prospective study from India demonstrated that the frequency of treatments for biofilm-negative endophthalmitis was significantly less than that for biofilm-positive endophthalmitis, and patients with biofilm-positive endophthalmitis had worse final visual acuity (8).

Biofilm is a complex bacterial aggregate wrapped in a self-generated matrix of extracellular polymeric substances, which is one of the key strategies for bacteria to survive unexpected changes in living conditions (9). The biofilm matrix includes extracellular polysaccharides, extracellular DNA (eDNA), proteins, and lipids, which account for more than 90% of biofilm biomass and act as scaffolds that adhere to biotic and abiotic surfaces, shielding bacteria in response to antibiotics and host immune responses (10). eDNA in biofilms facilitates virulence factor propagation and can be assembled into viscoelastic networks, increasing the stability of biofilms (11). The bacteria in the biofilm can evade the host immune response, and its resistance to antibiotic therapy is 1,000 times that of free bacteria (12).

As antibiotic resistance increases, 1.27 million people die each year from drug-resistant bacterial infections (13). Due to the ability to disrupt biofilms, antimicrobial peptides (AMPs) are considered promising drug candidates to combat multidrug-resistant bacteria (14). AMPs accumulate on the bacterial membrane by interacting with acidic substances or anionic molecules, thinning the membrane and forming transient pores, thereby disrupting its barrier function (15, 16). Antimicrobial peptides have a wide application prospect because of their antibacterial properties and membrane lysis mechanism. As a new type of antibacterial biomaterial, it is of great significance to explore the therapeutic effect of antimicrobial peptides on endophthalmitis.

**Fig 1:**
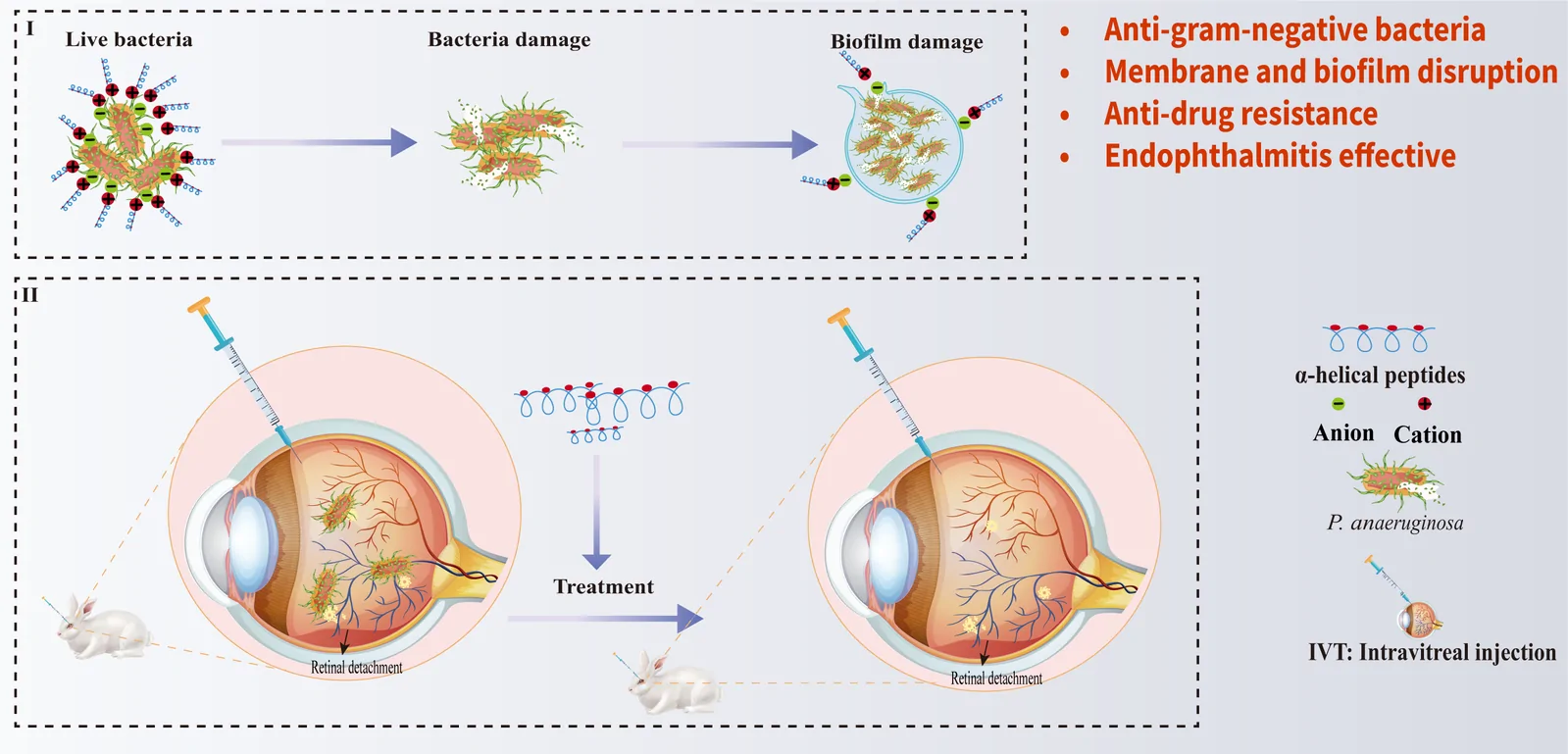
Part I: the antibacterial mechanism of helical peptides. Part II: the treatment strategy of AMPs in endophthalmitis.

Based on protein folding theory, non-natural helical peptides AMPn can be effectively synthesized. Yang et al. (17) synthesized a series of non-natural helical peptides: (LLKK)₃C, (LLKK)₂, and C(LLKK)₂C. Their advantage lies in effectively broadening the antibacterial spectrum of helical peptides by incorporating alcohol functional groups. Preliminary experiments demonstrated that (LLKK)_3_C and C(LLKK)_2_C adequately restrained the proliferation of *Candida albicans* and successfully treated *C. albicans* keratitis in mice, without causing toxicity to the ocular surface (18). Here, we investigated the antimicrobial mechanism and the therapeutic effect of AMPs against *P. aeruginosa* endophthalmitis (Fig. 1). We conducted minimum inhibitory concentration (MIC) tests against G− bacteria, *P. aeruginosa*, *Escherichia coli*, and *Klebsiella pneumoniae,* and G+ bacteria, *Staphylococcus aureus*. We investigated the antibacterial mechanism and membrane destruction mechanism of antimicrobial peptides. Additionally, we assessed the cytotoxicity of the drugs on ARPE-19, Müller cells, and rabbit eyes. Finally, we employed a *P. aeruginosa* endophthalmitis model to assess the antibacterial ability of peptides compared with conventional antibiotics.

## RESULTS

### (LLKK)_3_C and C(LLKK)_2_C show antimicrobial effectiveness in *P. aeruginosa*

**Fig 2:**
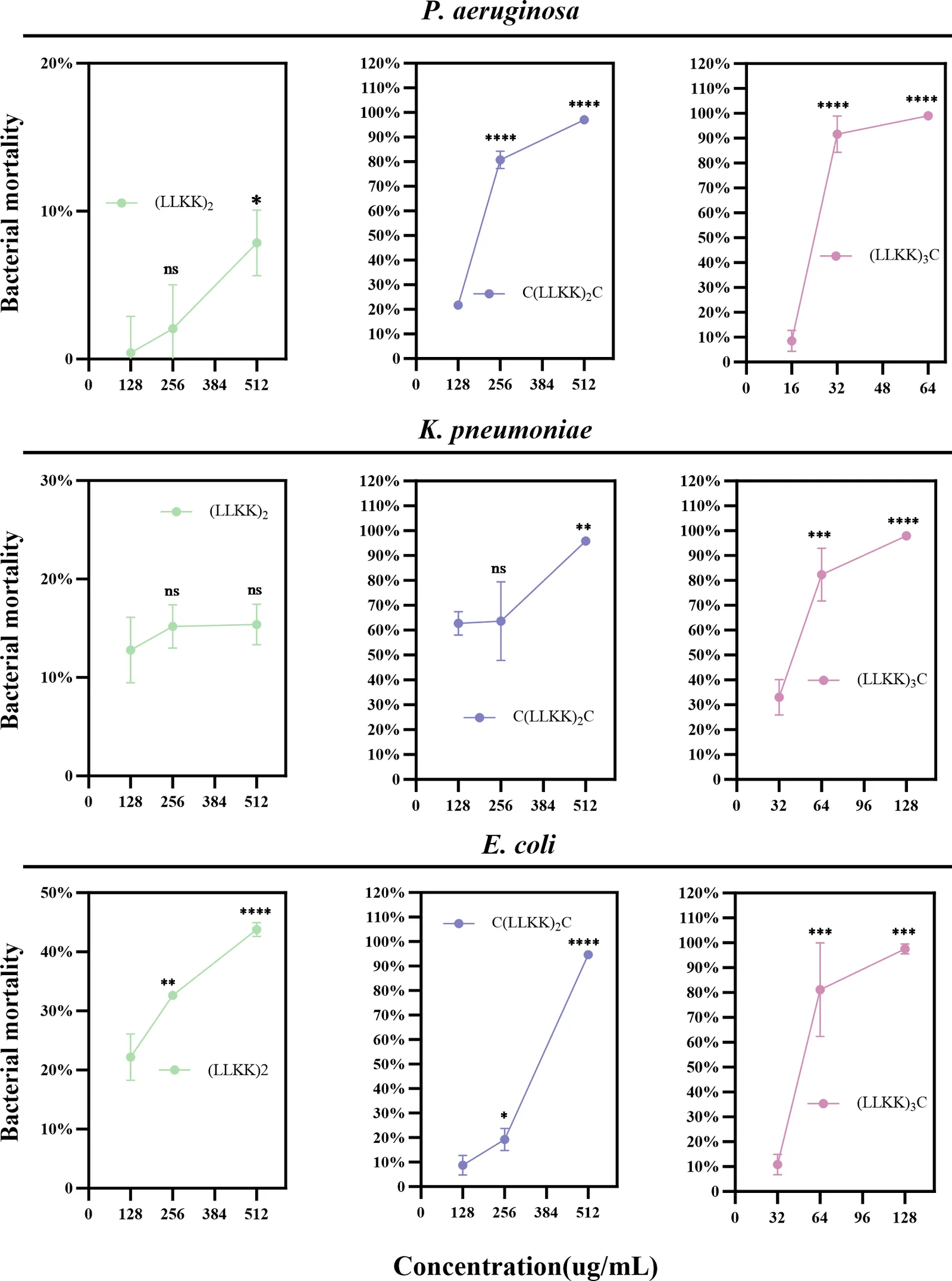
Inhibition rate of AMPs at different concentrations. One-way ANOVA analysis. ns: not significant, *P < 0.05, **P < 0.005, ***P < 0.001, ****P < 0.0001.

AMPs (LLKK)_3_C and C(LLKK)_2_C displayed significantly enhanced activity against G− bacteria than (LLKK)_2_ (Table 1). MIC is defined as the lowest concentration that prevents bacterial growth (18). We examined the MIC of peptides and amikacin against both G+ and G− bacteria. (LLKK)_2_, (LLKK)_3_C, and C(LLKK)_2_C had weak effects against *S. aureus* (MIC > 512 µg/mL). Among them, (LLKK)_3_C was most effective against gram-negative bacteria. The MICs of (LLKK)_3_C are 64–128 μg/mL (*K. pneumoniae*), 128 μg/mL (*E. coli*), and 32 μg/mL (*P. aeruginosa*). And the MICs of C(LLKK)_2_C are 512 μg/mL (*K. pneumoniae*), 512 μg/mL (*E. coli*), and 256 μg/mL (*P. aeruginosa*). While the MICs of (LLKK)_2_ are >512 µg/mL (*K. pneumoniae, P. aeruginosa,* and *E. coli*). As shown in Fig. 2, the MIC_90_ of C(LLKK)_2_C is >512 µg/mL (*K. pneumoniae, P. aeruginosa,* and *E. coli*), and the MIC_90_ of (LLKK)_3_C is at 128 µg/mL (*K. pneumoniae* and *E. coli*). *P. aeruginosa* is more sensitive to (LLKK)_3_C as the MIC_90_ is at 32 μg/mL. With the increase in concentration, there is a significant difference between the antibacterial rate at low concentrations and that at high concentrations.

**TABLE 1:** Minimum inhibitory concentration

Drugs	MIC (μg/mL)				
	G+		G−		
	*S. aureus* ATCC 43300	*S. aureus* ATCC 6538	*K. pneumoniae* ATCC 700603	*P. aeruginosa* ATCC 15442	*E. coli* ATCC 25922
(LLKK)_2_	>512	>512	>512	>512	>512
C(LLKK)_2_C	>512	>512	512	256	512
(LLKK)_3_C	512	>512	64−128	32	128
Amikacin	–^*a*^	–	2	1	8
Colistin	–	–	2	2	1

^*a*^ “–” represents no relevant data available.

### (LLKK)_3_C exhibits sustained potency against *P. aeruginosa* without resistance development

**Fig 3:**
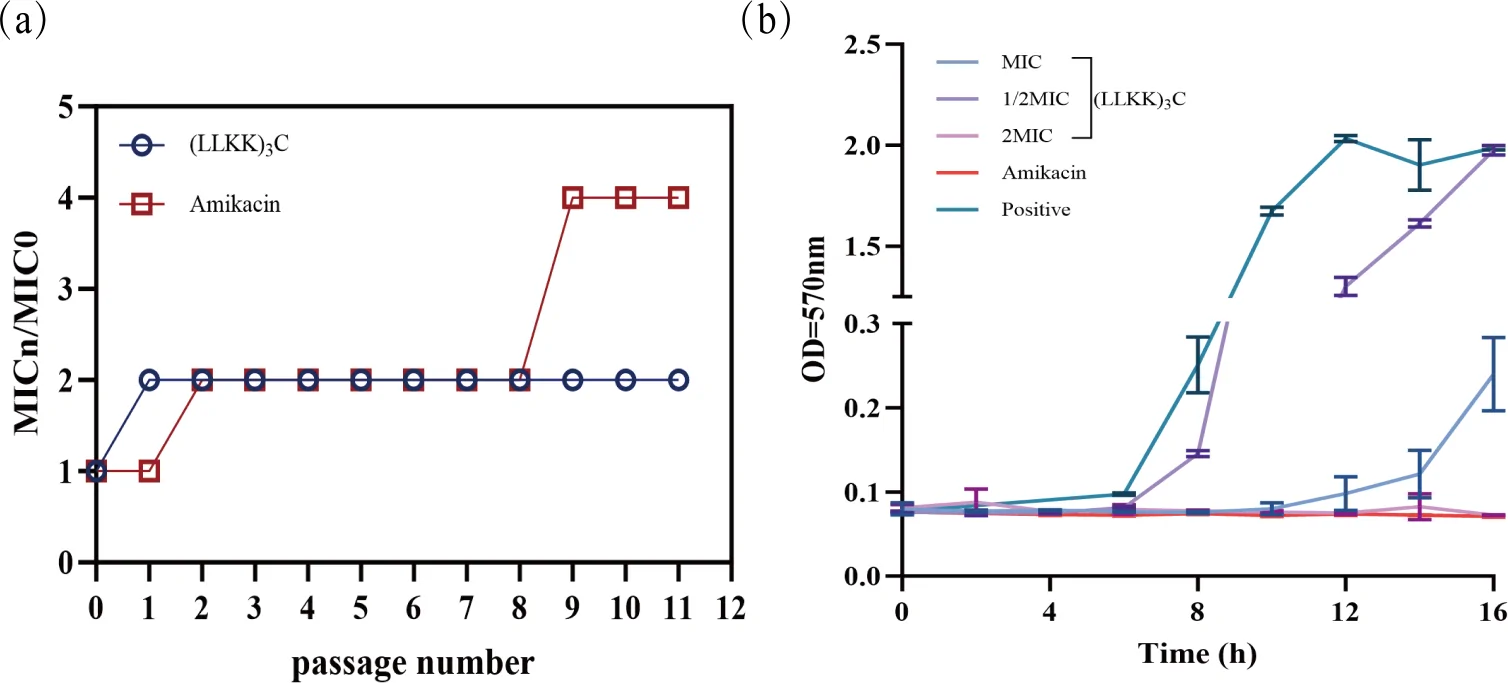
(**a**) Changes in MICs of (LLKK)_3_C and amikacin against *P. aeruginosa*. (**b**) Time-kill kinetics of (LLKK)_3_C at 1/2× MIC, MIC, and 2× MIC and of amikacin at MIC.

The resistance of (LLKK)_3_C is more stable than that of Amikacin in *P. aeruginosa*. Serial passage resistance selection assays demonstrated stable MIC values of (LLKK)_3_C (64 μg/mL) against *P. aeruginosa* over 10 consecutive passages (Fig. 3a). In contrast, amikacin-treated populations exhibited progressive MIC increases: a twofold rise from 1 to 2 μg/mL by passage 2, culminating in a fourfold increase by passage 9. Time-kill kinetics at respective MIC concentrations (Fig. 3b) revealed comparable bactericidal activity between (LLKK)_3_C and amikacin, both achieving ≥99.9% viability reduction within 24 h. In addition, we examined the stability of (LLKK)_3_C in an *in vitro* simulated physiological environment (Fig. S1 is available at https://doi.org/10.5281/zenodo.19666117). The results showed that at the MIC concentration, (LLKK)3C gradually decomposed over time. After incubation at 37°C for 24 h, the residual concentration of (LLKK)_3_C was 19.0738 μg/mL (58.9%).

### C(LLKK)_2_C and (LLKK)_3_C disrupt *P. aeruginosa* biofilms via membrane targeting

**Fig 4:**
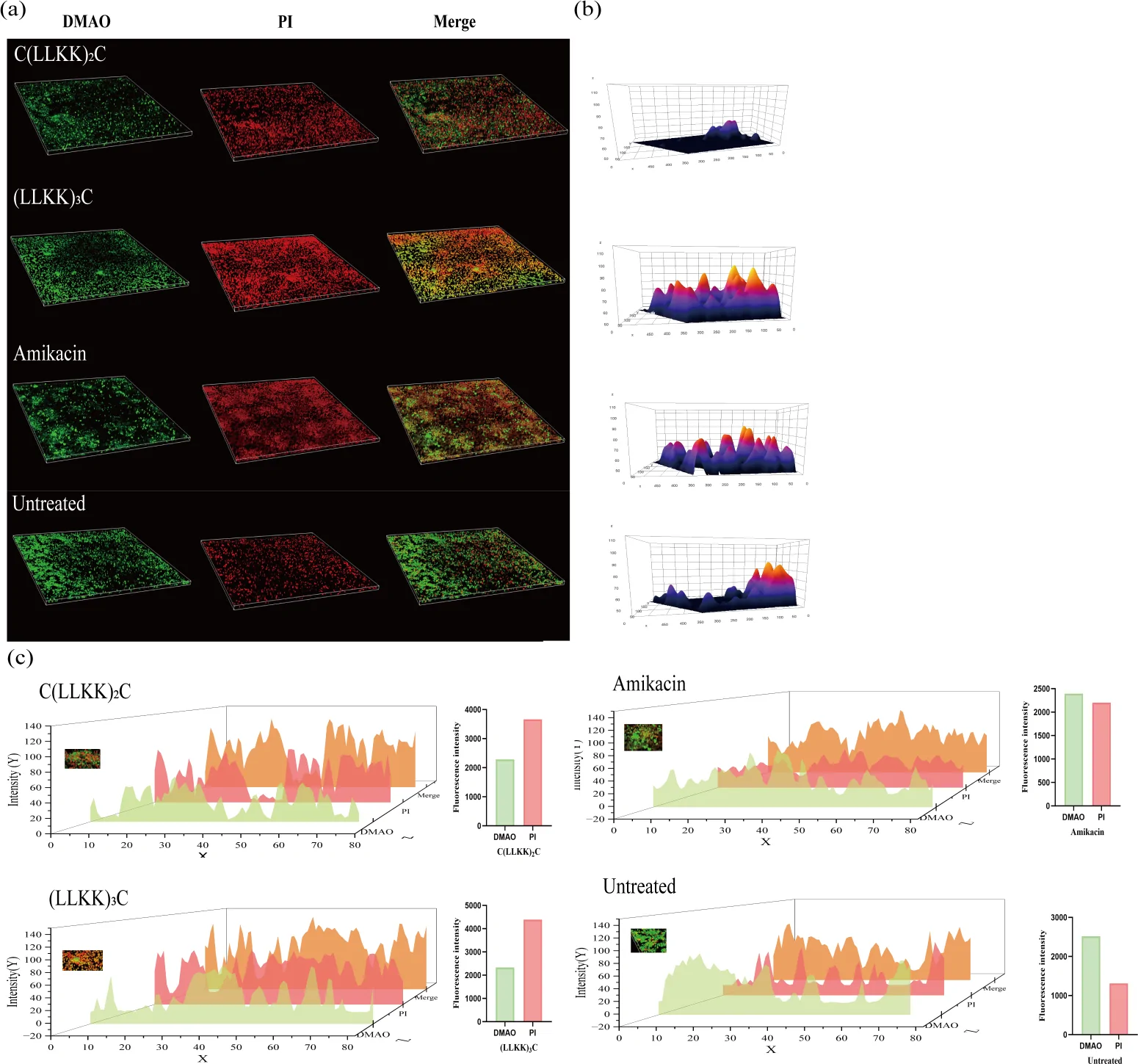
(**a**) Confocal laser scanning microscopy images of *P. aeruginosa*. Living cells were stained green, and dead cells were stained red. Biofilms were observed under the 63× oil microscope. The scale bar: 1 μm. (**b**) Surface plot of the merged imaging. (**c**) Local fluorescence intensity analysis (plot profile).

Biofilm is one of the main factors of drug resistance in bacteria. We examined the effect of peptides on biofilm destruction of *P. aeruginosa* and the mechanism of destruction. 3D laser confocal microscopy (CLSM) can detect the thickness of biofilm through Z-stacking, so we used CLSM to monitor the results of biofilm disruption (Fig. 4). DAMO green fluorescence labeled live cells, while PI red fluorescence labeled dead cells of the biofilms. After 24 h of incubation, the strains were apparently aggregated on the coverslips (Fig. 4a). In the untreated group, the majority of cells were in green, while the minority were in red. In a group of C(LLKK)_2_C, (LLKK)_3_C, and amikacin, the predominant was the red aggregation of dead cells, and green living cells were less than dead cells. Subsequently, we analyzed the overall fluorescence intensity and the local fluorescence distribution with ImageJ. The overall fluorescence intensity indicates the number of bacteria remaining on the fragment (Fig. 4b). In the area of bacterial aggregation of C(LLKK)_2_C and (LLKK)_3_C, the green fluorescence was less than the red fluorescence, indicating that the polypeptide can effectively destroy the biofilm (Fig. 4c).

**Fig 5:**
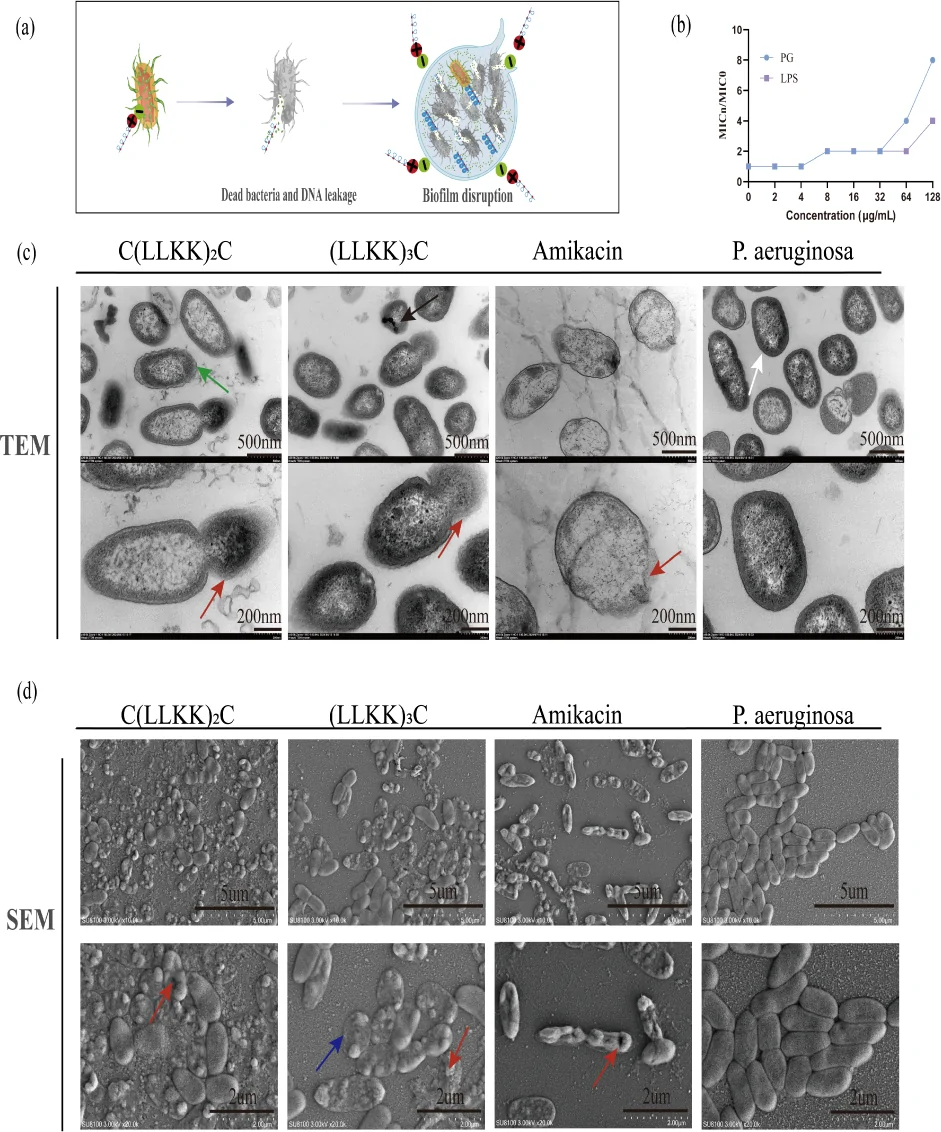
(**a**) C(LLKK)2C and (LLKK)3C combined with DNA and membrane cracking to damage the bacteria and biofilm. (**b**) Increased concentrations of PG and LPS can affect the MIC of (LLKK)3C. (**c**) TEM was used to show the changes in bacterial membrane morphology after treatment with C(LLKK)2C, (LLKK)3C, amikacin, and the untreated control group, respectively, for 24 h post-treatment with *P. aeruginosa*. Red arrow: transudation; green arrow: radiant changes and pigmentation; black arrow: chromatin condensed; white arrow: intact bacteria. (**d**) The SEM images of the bacterial structure. Red arrow: holes; blue arrow: bacterial rupture.

Furthermore, we explored the mechanism of peptides to disrupt the bacterial membrane and biofilms (Fig. 5a). We used TEM and SEM to observe changes in biofilm and bacterial membrane morphology in response to peptides. After 24 h of incubation of C(LLKK)_2_C, (LLKK)_3_C, and amikacin, a porous membrane can be seen on the surface of the bacterial cells with leakage of contents (Fig. 5c). The *P. aeruginosa* in the untreated group was tightly linked together through the biofilm, as observed by SEM. While in the treated group, the bacterial arrangement was sparse and irregular, and the biofilm was destroyed, and concave holes and bacterial rupture can be seen on the bacterial surface (Fig. 5d). TEM and SEM provided direct evidence of bacterial membrane disruption and biofilm destruction, confirming the antibacterial effects of C(LLKK)_2_C and (LLKK)_3_C. Subsequently, we determined the target of (LLKK)_3_C action on the cell membrane. LPS and phosphatidylglycerol (PG) are the main components of the cell membrane of G− bacteria. When LPS concentration was gradually increased from 0 to 128 μg/mL, the MIC of (LLKK)_3_C against *P. aeruginosa* was increased to four times (range 32 to 128 μg/mL). When the concentration of PG was gradually increased from 0 to 128 μg/mL, the MIC of (LLKK)_3_C against *P. aeruginosa* was increased to eight times (range 32 to 256 μg/mL). This suggests that LPS and PG had a significant effect on the antimicrobial activity of (LLKK)_3_C, which exerts membrane cleavage by acting on LPS and PG.

**Fig 6:**
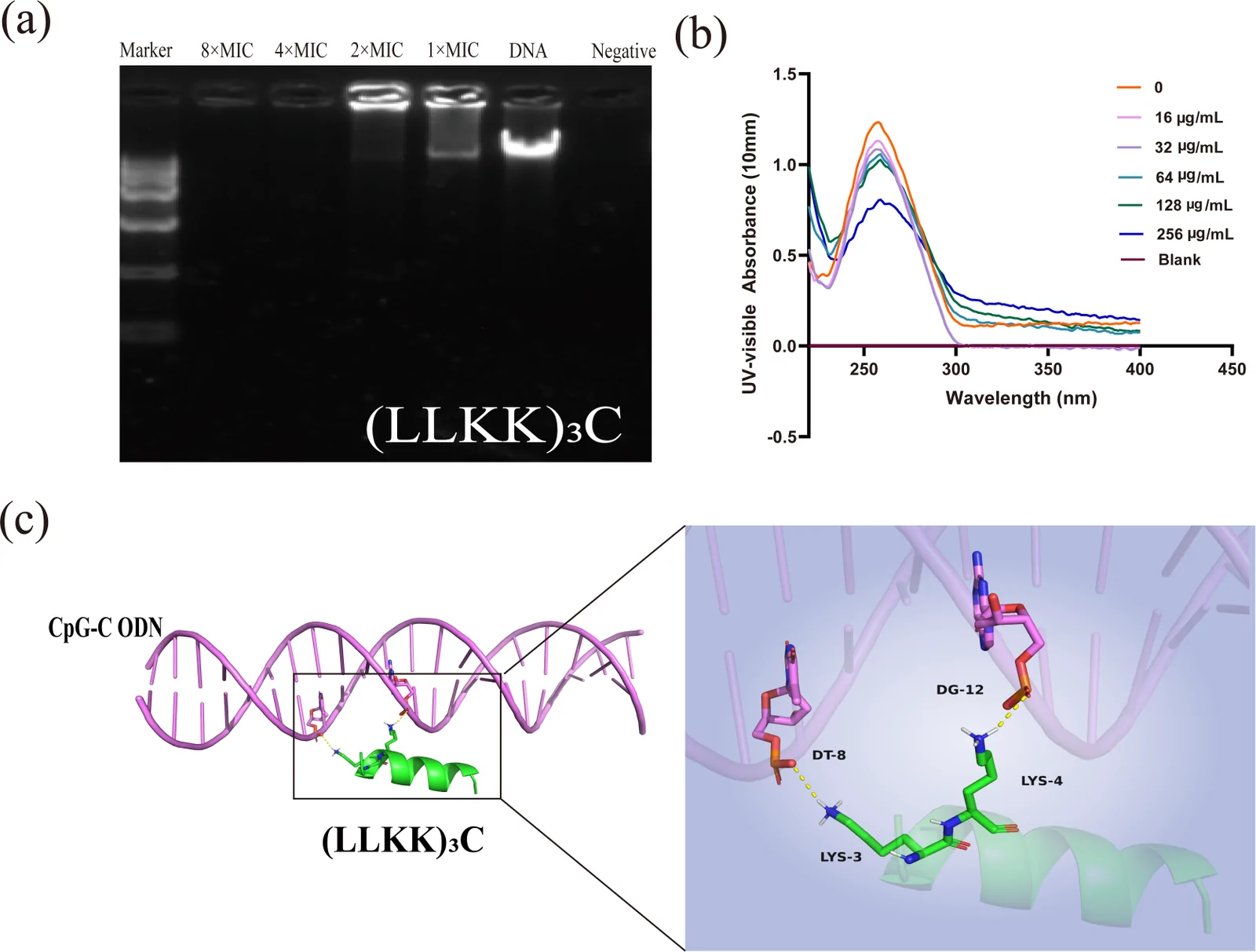
(**a**) DNA gel electrophoresis assay. (**b**) UV absorbance at 220–400 nm. (**c**) AlphaFold3 predicts the interaction of (LLKK)3C and CpG-C ODN.

DNA can spread the virulence factors and trigger the inflammatory response; moreover, it helps stabilize the biofilm (11, 19). As shown in the DNA gel electrophoresis assay, (LLKK)_3_C could combine with the DNA of *P. aeruginosa* at different concentrations (Fig. 6a). The absorbance peak of DNA was observed at 260 nm. With the increasing peptide concentration, the maximum DNA peak was gradually decreased (Fig. 6b). To clarify the binding mechanism of (LLKK)_3_C, we used AlphaFold3 to model its interaction with unmethylated class-C CpG oligodeoxynucleotides (CpG-C ODNs), which are minimal immunostimulatory motifs in bacterial DNA. As shown in Fig. 6c, the lys3 and lys4 structures of helical peptide (LLKK)_3_C interacted with T8 and G12 sites of DNA via DIH (D—hydrophobic interaction, I—electrostatic interaction, H—hydrogen bonding). Our results, demonstrated by EMSA and UV spectroscopy, and supported by structural modeling, show that DNA binds to (LLKK)_3_C.

### Biocompatibility

**Fig 7:**
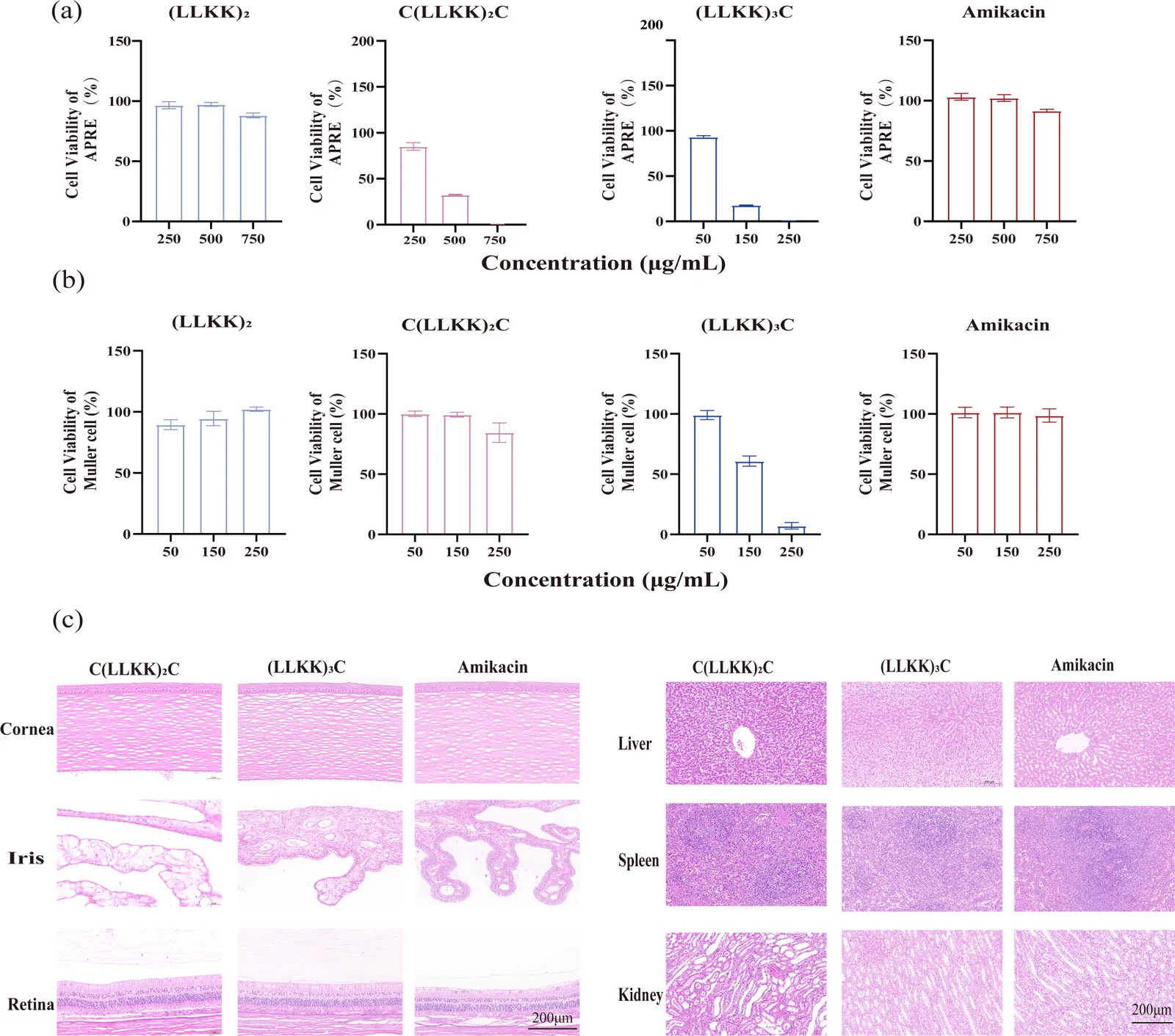
Evaluation of antimicrobial peptides using *in vitro* and *in vivo* methodologies. (**a**) The cytotoxicity of AMPs and amikacin on ARPE cells. (**b**) The cytotoxicity of AMP and amikacin on Müller cells. (**c**) HE staining of eye tissue. HE, liver, spleen, and kidney. Scale bar: 200 μm.

Biocompatibility is critical for the clinical utilization of biomedical materials. The biocompatibility of three antimicrobial peptides and amikacin was assessed in ARPE and Müller cells using the CCK-8 assay (Fig. 7a and b). As shown in Fig. 7a, (LLKK)_3_C and C(LLKK)_2_C exhibited higher toxicity at lower concentrations than (LLKK)_2_ and amikacin. The IC_50_ of (LLKK)_3_C for ARPE and Müller cells ranged between 50 and 150 μg/mL, whereas that of C(LLKK)_2_C for ARPE was between 250 and 500 μg/mL. Increased peptide length and helicity appeared to correlate with higher biological toxicity. *In vivo* toxicity was evaluated in New Zealand white rabbits, which were divided into four groups of two rabbits each. The drug was injected into the vitreous cavity twice with an interval of 1 day [C(LLKK)_2_C: 7 μg/mL, (LLKK)_3_C: 4 μg/mL, amikacin: 4 μg/mL]. The pathological results of cornea, iris, and retina were analyzed. The cornea of amikacin and (LLKK)_3_C was slightly thicker than that of C(LLKK)_2_C, but the corneal structure was complete, and the cells were dense. Iris and retina were intact, and no toxicity was observed. No obvious toxicity was found in the liver, spleen, or kidney.

### Treatment effect of endophthalmitis

**Fig 8:**
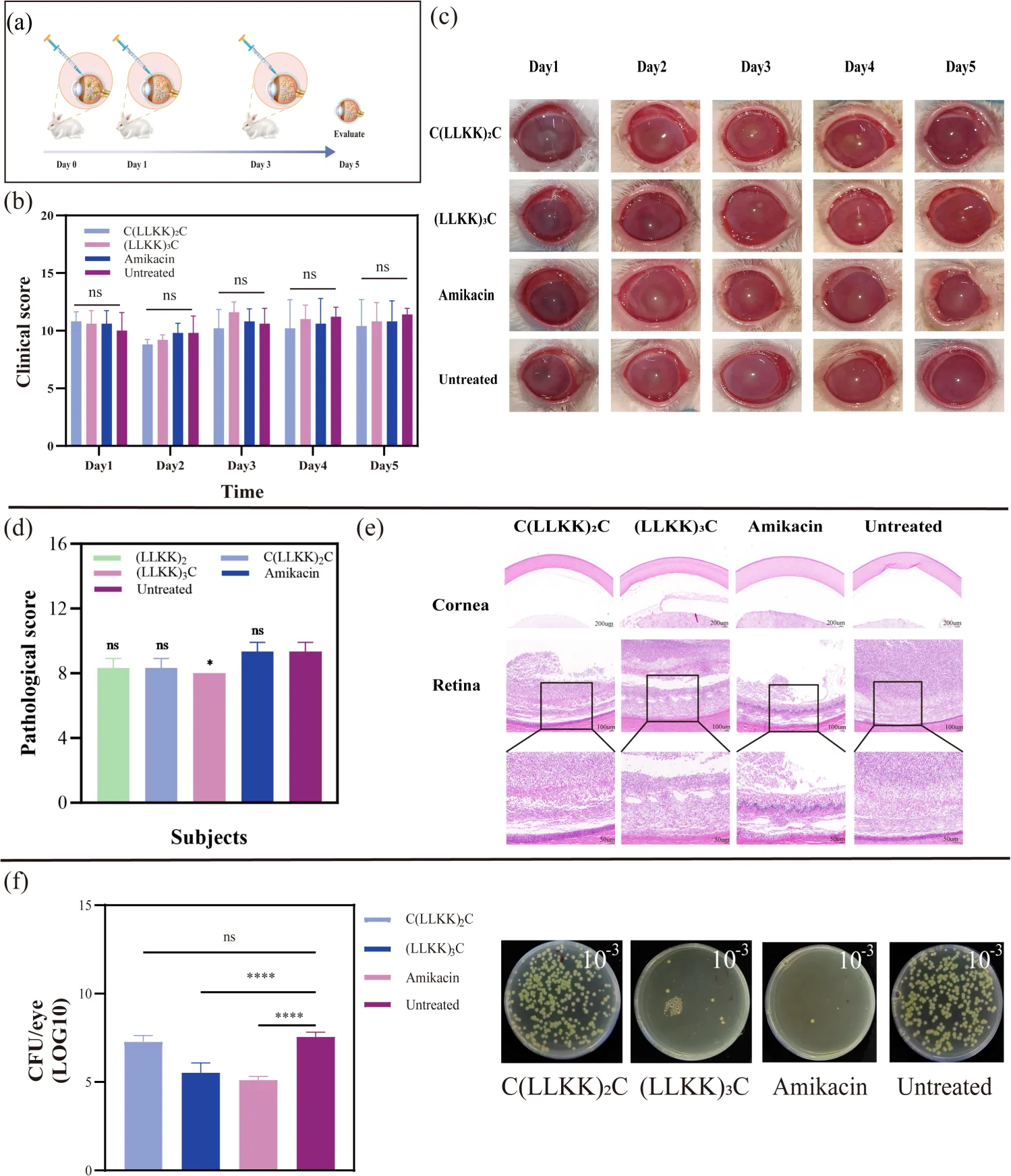
Treatment of the rabbit endophthalmitis model. (**a**) Modeling and therapeutic evaluation of endophthalmitis. (**b** and **c**) Macroscopic appearance and clinical scoring of the eye from day 1 to day 5. (**d** and **e**) Pathological scoring of the eyes from each group. (**f**) Eye grinding bacterial count analysis on day 5. In the amikacin-treated group, a partially intact retina is seen, whereas (LLKK)3C treatment has led to retinal destruction and choroidal vascular hyperplasia. Other groups exhibit full-thickness retina destruction and significant inflammatory cell infiltration in the vitreous cavity. All groups show inflammatory exudates, fibrotic changes in the anterior chamber, and corneal edema. Pathological scoring was independently performed by two clinically experienced physicians. One-way ANOVA analysis. ns: not significant, *P < 0.05, **P < 0.005, ****P < 0.0001.

The therapeutic effects of peptides and amikacin in a rabbit endophthalmitis model were assessed using clinical scoring (Fig. 8b and c). Daily changes in each group of eyes were recorded and scored, with no significant differences observed between groups. In the untreated group, there were signs of conjunctival edema, increased secretions, corneal opacity, anterior chamber pus, and total retinal destruction with extensive inflammatory cell infiltration. After two doses of (LLKK)_3_C and amikacin, conjunctival congestion was slightly relieved, with localized corneal edema and unclear fundus visibility. Bacterial counts in treated groups were significantly lower than in the untreated group (*P* < 0.05) (Fig. 8f). Based on the pathological results, a significant infiltration of inflammatory cells was observed in the retina and vitreous cavity (Fig. 8e), resulting in irreversible damage to the retinal structure. Although both (LLKK)_3_C and amikacin demonstrated good antibacterial effects, they were unable to prevent the inflammatory cascade triggered by endophthalmitis. Bacterial counts after treatment showed that the (LLKK)_3_C and amikacin-treated groups significantly reduced the bacterial load in the eyeball compared with the untreated group [(LLKK)_3_C: 5.532 ± 0.5521; amikacin: 6.116 ± 0.1979; untreated: 7.552 ± 0.2701; *P* < 0.05]. The progression of endophthalmitis was further analyzed through histopathological examination of eye sections, where a large infiltration of inflammatory cells was seen in both retinal and vitreous tissues across all groups (Fig. 8d and e). In the amikacin-treated group, part of the retina was preserved, corneal edema was mild, and iris inflammation was less severe. However, in the C(LLKK)_2_C, (LLKK)_3_C, and (LLKK)_2_ groups, there was complete retinal destruction and diffuse corneal edema with significant inflammatory infiltration. (LLKK)_3_C had a lower pathological score of eyeball, which was significantly different from the untreated group (*P* < 0.05), and there was no significant difference between the other groups and the untreated group. These results suggest that antibacterial therapy alone may not be sufficient to prevent retinal damage, and combined anti-inflammatory treatment may be necessary to manage infections caused by *P. aeruginosa*.

## DISCUSSION

AMPs play a crucial role in bacterial eradication and biofilm disruption. The helical structure of AMPs enhances their bactericidal ability while reducing bacterial resistance. In our research, we have detected the antibacterial ability and the mechanism of the three AMPs. We have found that (LLKK)_3_C exhibited excellent antibacterial function in the three helical peptides, and *P. aeruginosa* is more difficult to develop resistance to (LLKK)_3_C. The introduction of sulfhydryl groups to the spiral peptides virtually improved its antibacterial efficacy (17). Cysteine substitution at the C and N termini of (LLKK)_2_ resulted in C(LLKK)_2_C, which had increased helicity and improved interaction with biofilms. Furthermore, increasing the peptide length, as seen in (LLKK)_3_C, also enhanced its antibacterial activity. These results indicate that both C(LLKK)_2_C and (LLKK)_3_C exhibited superior antibacterial activity compared with (LLKK)_2_, particularly against *P. aeruginosa*.

Our results illustrated that (LLKK)_3_C could cleave the bacterial membrane and allow the content to flow out. Biofilms, which are structured microbial communities attached to surfaces, provide a diffusion barrier through their polysaccharide matrix, limiting the penetration of antimicrobial agents and contributing to bacterial resistance (20). *P. aeruginosa* biofilms can increase antibacterial resistance by 10,000-fold (21). These pieces of evidence strongly indicate that disrupting mature biofilms is crucial in combating bacterial resistance. The mechanism of action of antimicrobial peptides involves pore formation and membrane disruption (17). When the binding capacity of cationic peptides to anionic biofilms exceeds that of the biofilm matrix, antimicrobial peptides can penetrate the biofilm barrier and lyse the embedded bacteria (22). The surface of the bacterial membrane was perforated, and the biofilm was destroyed. Moreover, (LLKK)_3_C could target PG and LPS on the bacterial membrane, which proved the membrane destruction effect of (LLKK)_3_C. Furthermore, (LLKK)_3_C could be combined with the DNA to block the spread of virulence factors. This study only demonstrates that antimicrobial peptides exert their antibacterial effects by binding to DNA; however, the mechanism of interaction between DNA and antimicrobial peptides requires further experimental investigation.

In a rabbit model of endophthalmitis, both (LLKK)_3_C and amikacin showed promising therapeutic effects. Although amikacin is a second-line treatment for the clinical treatment of G− bacterial endophthalmitis, it is the preferred choice for patients allergic to ceftazidime. However, both amikacin and (LLKK)_3_C failed to completely suppress the inflammatory response in the vitreous cavity, and (LLKK)_3_C did not have a direct inflammatory regulation effect (17), suggesting that future treatments for endophthalmitis should combine antibacterial and anti-inflammatory effects. Host defense peptides with dual antimicrobial and anti-inflammatory functions may hold promise for the treatment of endophthalmitis.

This study demonstrated that (LLKK)_3_C inhibits the growth of *P. aeruginosa* by penetrating biofilms, combining DNA, and killing bacteria through biofilm disruption. In this era of antibiotic resistance, as *P. aeruginosa* is tricky in the treatment of infections, (LLKK)_3_C might be a promising antibacterial agent in the future. However, (LLKK)_3_C showed significant cytotoxicity in this study. Subsequent studies should prioritize rational optimization to reduce toxicity while preserving activity, thereby improving prospects for translation.

## MATERIALS AND METHODS

### Bacterial strains and minimal inhibitory concentration

Standard bacterial strains were stored in the laboratory: *S. aureus* (ATCC 43300 and ATCC 6538), *P. aeruginosa* (ATCC 15442), *K. pneumoniae* (ATCC 700603), and *E. coli* (ATCC 25922). GL Biochem (Shanghai, China) synthesized the (LLKK)_3_C, C(LLKK)_2_C, and (LLKK)_2_ and confirmed the purity (over 95%) by reverse-phase HPLC. The MICs were determined using the broth dilution method. The bacterial suspensions were modified to a 0.5 McFarland standard (DensiCHEK turbidimeter, bioMérieux) and further diluted to 10^5^ CFU/mL. Add 100 μL of bacterial suspension to 100 μL of the peptide/antibiotic solution in each well. The plates were incubated at 37°C for 16–18 h, after which absorbance was measured at OD 570 nm. Calculate the bacterial mortality as follows:


$$Bacterial\;mortality=[(OD570_{Positive}-OD570_{Experimental})/(OD570_{Positive}-OD570_{Negative})]\times 100\%.$$

### Drug resistance test

The bacteria that survived at the concentration of 1/2× MIC for 11 generations were continuously taken for MIC, and the change of MICn/MIC0 was calculated.

### Mechanism of membrane destruction

Culture *P. aeruginosa* biofilms on round coverslips for 24 h. Wash the coverslips twice with PBS. Add (LLKK)_3_C, C(LLKK)_2_C, and amikacin at 4× MIC concentrations, then culture for 24 h. Wash the coverslips twice to remove residual culture medium. Following the DMAO/PI Bacterial Staining Kit protocol (C2030S, Beyotime Biotechnology, China), stain coverslips for 20 min. Mount the coverslips and observe biofilms using CLSM (Nikon Eclipse Ti2, Japan). ImageJ was used to analyze the results.

SEM observation: culture a bacterial suspension (1 × 10⁸ CFU/mL) on 1 × 1 cm² coverslips at 37°C for 24 h. After biofilm formation, remove the bacterial solution and wash the biofilm three times with PBS. Treat the samples with peptides (256 μg/mL) and culture them at 37°C for 24 h. Wash the coverslips three times with PBS. Dehydrate them using ethanol solutions (30%, 50%, 70%, 90%, and 100%; 10 min each). Air-dry samples for 30 min at room temperature, then coat them with gold for conductivity. Analyze the samples using SEM.

For TEM detection, we performed the following steps. First, culture a bacterial suspension (1 × 10⁸ CFU/mL) in 6-well plates at 37°C for 24 h. After biofilm formation, wash the wells three times with PBS. Treat the biofilm with peptides (256 μg/mL) and culture at 37°C for 24 h. Scrape the biofilm into a centrifuge tube. Centrifuge at 10,000 rpm, resuspend the pellet, and fix it in 2.5% glutaraldehyde solution (R20515, Shanghai Yuanye Bio-Technology). Prepare ultrathin sections using a Leica EM UC6 microtome (Leica, Germany). Stain sections with uranyl acetate for 10 min and lead citrate for 20 min.

Phosphatidylglycerol and LPS are key components of bacterial membranes. Using the checkerboard method, we mixed twofold gradient concentrations of phosphatidylglycerol and LPS with (LLKK)_3_C. This mixture was combined with a bacterial solution (1 × 10⁵ CFU/mL) and incubated for 18 h. We then measured changes in MIC values.

### DNA gel electrophoresis and DNA binding assays

We performed the test following Hou et al. (23). First, extract *P. aeruginosa* DNA using a Bacterial DNA Kit (D3350-01, Omega Bio-Tec, USA) and store at −20°C. Dilute the genomic DNA to 30 ng/μL with wash buffer. Mix 4 μL DNA with 16 μL buffer in eight tubes and incubate for 1 h. Prepare peptide solutions at MIC, 2× MIC, 4× MIC, and 8× MIC concentrations. Load 5 μL of each mixture into a 0.7% agarose gel. Run electrophoresis at 110 V for 23 min using a 1.5 kb DNA marker. Capture images with a gel imaging system (QuickGel 6100, Monad, China).

Peptide solutions were added at final concentrations of 1/2× MIC, MIC, 2× MIC, 4× MIC, and 8× MIC, respectively. DNA was adjusted to a final concentration of 50 ng/μL, and the blank group was 1× TE. The absorbance spectra were determined using a NanoPhotometer (Implen, Germany) in the 220–400 nm range.

### Prediction of (LLKK)_3_C and DNA interaction sites

AlphaFold3 is a tool used to predict protein structures and the binding sites of proteins to DNA or RNA. AlphaFold3 showed some superiority in predicting the 3D structure of protein-protein, protein-ligand, and protein-nucleic acid complexes (24). In this study, we used AlphaFold3 to predict the interaction mode and site of C(LLKK)_2_C and (LLKK)_3_C on CPG-C ODN (sequence TCGTCGTTTTCGGCGCGCGCCG unmethylated class C CpG oligodeoxynucleotides) (25). CpG-C ODN is the smallest unit in the bacterial genome that has immunostimulating effects. Amino acid sequence and DNA sequence were submitted to the AlphaFold3 server to predict five interaction models, and PyMOL was used to draw maps to show specific interaction amino acid sites (26).

### Cell Counting Kit-8 assay

#### ARPE-19 cell viability assay

Culture ARPE-19 cells (GNHu45, Stem Cell Bank, Chinese Academy of Science) in DMEM (Corning, USA) containing 10% FBS. Seed 5 × 10⁵ cells/mL per well in a 12-well plate and incubate at 37°C with 5% CO₂ for 12 h. Add peptides or antibiotics at varying concentrations to designated wells and incubate for 24 h. Wash twice with PBS. Add serum-free DMEM with 10% CCK-8 reagent (C0038, Beyotime Biotechnology, China) and incubate for 2 h. Measure absorbance at 450 nm.

#### MIO-M1 cell viability assay

Culture the human Müller cell line MIO-M1 (BFN6021542, Cell Bank, Chinese Academy of Sciences) in RPMI 1640 (Corning, USA) containing 10% FBS. Seed 5 × 10⁴ cells/well in a 96-well plate and incubate at 37°C with 5% CO₂ for 24 h. Add peptides or antibiotics at varying concentrations to designated wells and incubate for 12 h. Wash twice with PBS. Add serum-free RPMI 1640 with 10% CCK-8 reagent and incubate for 2 h. Measure absorbance at 450 nm.

### *In vivo* antibacterial properties

#### Endophthalmitis model

Twenty-five male New Zealand white rabbits (2 kg, 6–8 weeks old) were randomly assigned to five groups (five per group). The rabbits received food and water freely. All animal procedures followed ethical guidelines approved by the institute’s Ethics Committee (IACUC of AMMS-11-2024-022).

*P. aeruginosa* ATCC 15442 was cultured for the endophthalmitis model. Rabbits were anesthetized with isoflurane and one drop of proxymetacaine hydrochloride eye drops. A bacterial suspension (50 μL, 50 CFU/eye) adjusted to 1,000 CFU/mL was injected into the right vitreous cavity. Acute endophthalmitis developed within 24 h (day 1). Treatments started 24 h post-infection. Treatment groups (intravitreal injection, right eye): C(LLKK)_2_C: 100 μL of 7 mg/mL, repeated on day 3; (LLKK)_3_C: 100 μL of 4 mg/mL, repeated on day 3; Amikacin: 100 μL of 4 mg/mL, repeated on day 3; untreated group: no intervention. Dosages were based on preliminary data and clinical standards.

### Clinical score of endophthalmitis

The clinical severity of endophthalmitis was assessed daily from day 1 to day 5, following the standard proposed by Peyman et al. (27). Day 1 was designated as the day of infection, and day 2 as the day of drug injection. Clinical evaluations continued for 3 days post-treatment, until day 5.

### Bacterial load in the eyeball

Rabbits were euthanized via anesthesia overdose, and the entire eyeball was extracted and homogenized using an automatic freezing grinder (Shanghai Jingxin Industrial Development Co., Ltd.) at 4°C. A 10-fold serial dilution of the tissue homogenate was prepared, and 100 μL of each dilution was plated onto BHI agar plates. Plates were incubated for 16–18 h, and bacterial colonies were counted. Each experiment was repeated three times.

### H&E staining and pathological score

Fix eyeball samples in fixation solution (G1109, Wuhan Servicebio Technology) for 72 h. Embed in paraffin and section. Perform hematoxylin-eosin (H&E) staining, then dehydrate and seal slides. Score the cornea, anterior chamber, vitreous body, and retina from 0 to 3 using the pathological scoring criteria of Saleh et al. (28). Capture images with Nikon Eclipse E100 and DS-U3 microscopes (Nikon, Japan) (Nikon Corporation, Japan).

### *In vivo* safety tests

For *in vivo* safety testing, the drug is injected into the vitreous cavity according to the therapeutic dosage and frequency. The eyeballs were harvested for pathological examination via H&E staining to assess tissue structure and identify any adverse changes.

### Statistical analysis

All statistical data were conducted with GraphPad Prism (10, GraphPad Software Inc., USA). Data were calculated and processed as means ± SD of three independent experiments. Using a one-way ANOVA test to analyze the data. Statistically significant *P* values are indicated in figures and legends.
